# Privacy-Preserving Ambient Sensing for Activities of Daily Living: Multimodal Radar–Thermal Human Activity Recognition and Smart Plug Appliance Recognition

**DOI:** 10.3390/s26165066

**Published:** 2026-08-10

**Authors:** Bilal Mohammed, Jordan J. Bird, Isibor Kennedy Ihianle, Martin Harris, Geoff Archenhold, Yangang Xing

**Affiliations:** 1 Integrated System Technologies, Staffordshire WS13 8RZ, UK; 2 Department of Computer Science, Nottingham Trent University, Nottingham NG11 8NS, UK; jordan.bird@ntu.ac.uk (J.J.B.);; 3 School of Architecture Design and the Built Environment, Nottingham Trent University, Nottingham NG1 4BU, UK

**Keywords:** human activity recognition (HAR), activities of daily living (ADLs), mmWave radar, thermal sensing, sensor fusion, non-intrusive load monitoring

## Abstract

Continuous monitoring of Activities of daily living (ADLs) requires sensing systems that are privacy-preserving, low-power, and robust to environmental variation. Ambient sensing technologies provide an alternative to RGB video and wearable devices, but individual sensing modalities exhibit characteristic limitations. Sparse mmWave radar provides strong motion sensitivity but limited posture detail, low-resolution thermal sensing preserves posture-related spatial information, and smart plug telemetry captures only appliance-mediated behavioural interaction. To address these limitations, this paper proposes a layered multimodal ambient-sensing framework comprising a sparse-track 24-GHz FMCW radar, a 32×24 low-resolution thermal sensor, and a Moko smart plug. It experimentally evaluates a radar–thermal HAR branch together with a separate smart plug appliance-recognition branch. The framework proposes three streams to enable continuous non-wearable monitoring while maintaining redundancy and reduced privacy exposure for intelligent-building and ambient assisted living environments. Radar and thermal streams are jointly evaluated on binary motion and four-class posture and activity recognition tasks collected across multiple environmental configurations using recording-grouped cross-validation, while the appliance stream is evaluated using per-plug telemetry from residential-grade appliances. The radar–thermal streams use a single-subject, fixed-placement dataset of binary-motion windows and four-class posture and motion windows collected across six furniture configurations. The separate intrusive load monitoring stream utilises smart plugs to classify appliances. Regarding binary motion recognition, radar $\left(F_{1,\mathrm{Transformer}}=0.882\pm 0.034\right)$ and thermal $\left(F_{1,\mathrm{XGBoost}}=0.870\pm 0.069\right)$ pipelines achieved similar macro F1 performance. On the four-class posture and activity recognition task, thermal features $\left(F_{1,\mathrm{thermal}}=0.775\pm 0.053\right)$ substantially outperformed radar $\left(F_{1,\mathrm{radar}}=0.609\pm 0.110\right)$. Weighted late fusion produced only modest descriptive gains. Separately, smart plug telemetry demonstrated strong appliance recognition performance using lightweight tree-based models suitable for constrained edge deployment. The results support a scoped redundancy argument. Sparse track-level radar carries gross motion, while low-resolution thermal sensing carries posture. The smart plug appliance monitoring extends the framework toward appliance-mediated instrumental activity of daily living (IADL) monitoring, with lightweight tree-based models achieving strong recognition performance under constrained edge deployment conditions. The findings support a layered multimodal sensing architecture for privacy-preserving ADL monitoring, where radar contributes motion-sensitive coverage, thermal sensing contributes posture-aware spatial context, and smart plug telemetry contributes appliance-level behavioural evidence within intelligent healthcare and ambient assisted living environments.

## 1. Introduction

Population ageing is reshaping both the objectives of digital healthcare and the role of the home in care delivery. Rather than functioning solely as a location where care is delivered, the home is increasingly being reconceptualised as an active sensing environment capable of supporting continuous assessment, early intervention, and safer ageing. From a digital health perspective [1], this transition reflects a broader shift from intermittent clinic-centred measurement toward remote, longitudinal, and lower-burden monitoring.

This frame of reference has direct relevance to activities of daily living (ADLs). ADLs and instrumental activities of daily living remain core markers of functional independence, progression of frailty, and the ability to stay at home safely. Therefore, research on smart living and ambient assisted living has converged on sensing systems that can observe routine, movement, posture, and risk in ordinary domestic spaces [2,3,4]. Privacy, interoperability, low maintenance burden, resource constraints, and cross-environment robustness are recurring bottlenecks in real homes [2,5,6]. Furthermore, no single ambient modality provides a sufficient account of daily living. Radar technology is well suited for presence detection, gross movement, gait-related dynamics, and fall-sensitive motion patterns, while remaining robust to darkness and some forms of occlusion [7,8,9]. Its main limitation is weak spatial detail for fine posture interpretation, particularly when commercial hardware exposes only vendor-processed kinematic outputs. Low-resolution thermal arrays occupy a different point in the privacy-detail tradeoff. They suppress identity and visible texture while maintaining enough body shape and heat distribution to support posture recognition, coarse activity analysis, and privacy-preserving fall detection [10,11,12]. Their limits appear in weak thermal contrast, heat clutter, and temperature-sensitive environments [11,12]. Power monitoring provides a third complementary layer. Through appliance usage and routine timing, non-intrusive load monitoring can reveal meal preparation, kettle use, sleep-related disturbance, and broader patterns of a domestic routine without direct observation of the resident [13,14,15]. However, electricity data cannot directly represent body motion, posture transition, gait quality, or hazardous events such as a fall.

Within intelligent building environments, these sensing modalities provide complementary and redundant forms of behavioural evidence that together support a more comprehensive understanding of activities of daily living (ADLs) while maintaining privacy within the home. Power sensing captures routine-level interaction with appliances and domestic infrastructure. Radar sensing captures motion-centred evidence of mobility and activity, while low-resolution thermal sensing contributes coarse spatial and posture context without exposing identity-related visual detail. These complementary sensing characteristics suggest that intelligent building research for healthy ageing should be framed as a layered multimodal sensing problem rather than a competition between individual modalities. Multimodal monitoring surveys further argue that the principal advantage of non-contact sensor fusion lies in this complementarity, where heterogeneous sensing modalities compensate for each other’s limitations and can be selectively deployed according to the privacy requirements of different rooms and monitoring tasks [16]. However, the privacy literature also highlights an important challenge: multimodal integration increases inferential power, meaning that privacy-preserving design cannot be achieved solely through the removal of RGB cameras. It additionally requires data minimisation, access control, and careful decisions regarding which data are processed locally and which are exposed to external systems [5].

The architectural literature extends this discussion from individual sensing modalities to integrated intelligent building systems. Active and healthy ageing platforms are still often deployed as isolated islands of technology rather than interoperable ecosystems, limiting reuse, scalability, and cross-platform service development [6]. An integrated AIoTES architecture proposed in [6] addresses this problem through layered interoperability, semantic mediation, and explicit security and privacy services, showing that an intelligent building framework must connect heterogeneous devices, data models, and applications rather than simply accumulate sensors. For ageing-in-place research, architecture is therefore not a secondary implementation issue. It is part of the scientific problem.

**Figure 1:**
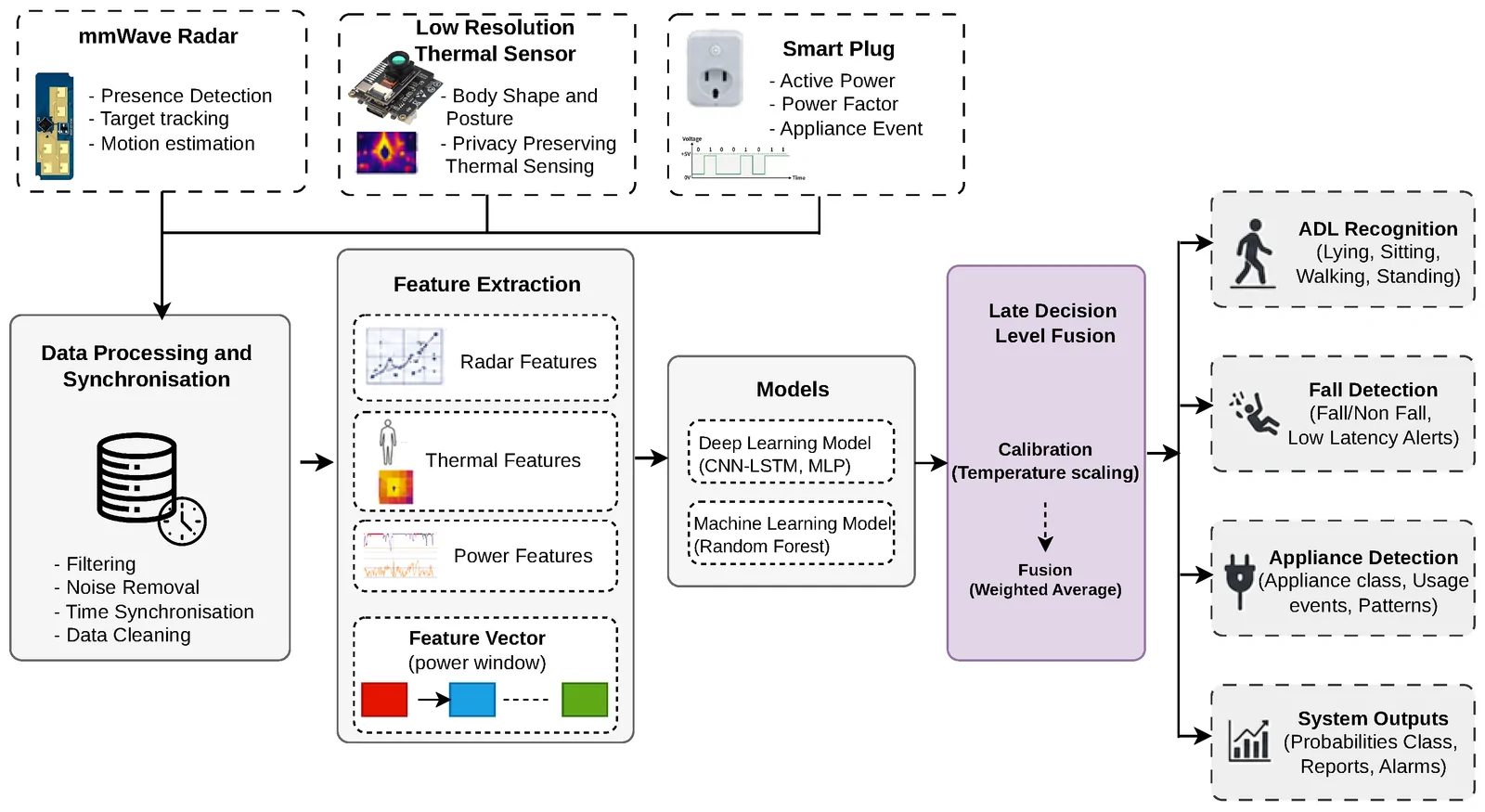
Proposed privacy-preserving multimodal architecture for ADL monitoring and appliance recognition using radar, low-resolution thermal sensing, and smart plug telemetry with late fusion.

This paper is motivated by these sensor and architectural knowledge gaps, investigating privacy-preserving ambient sensing for ADL-related recognition under explicit environmental variation while placing the resulting pipelines within a broader sentient building framework for healthy ageing. The experimental focus is on commercial LD2450 24-GHz radar (Shenzhen Hi-Link Electronic Co., Ltd., Shenzhen, China) and low-resolution thermal arrays collected across six laboratory environments. This design addresses two practical questions that remain under-explored in the literature: whether low-cost commercial radar and thermal sensing can support within-subject activity recognition across multiple room configurations under fixed sensor placement and whether radar–thermal late fusion provides additional descriptive value under a recording-grouped evaluation protocol. Power monitoring is treated here as a separate routine-level component of the broader framework, rather than as a direct substitute for resident-centred sensing, as seen in Figure 1.

The methods proposed by this work make the following scientific contributions:

- A privacy-preserving multimodal ambient-sensing framework for ADL monitoring combining sparse-track mmWave radar, low-resolution thermal sensing, and smart-plug appliance telemetry as complementary sensing streams within an intelligent-building context;
- An empirical evaluation of low-cost LD2450 24-GHz radar (Shenzhen Hi-Link Electronic Co., Ltd., Shenzhen, China) and MLX90640 thermal sensing (Melexis Technologies NV, Limburg, Belgium) for within-subject activity recognition across multiple room configurations, using recording-grouped cross-validation to control leakage between training and held-out recordings;
- An analysis of radar–thermal complementarity through unimodal and exploratory decision-level late-fusion pipelines, comparing their relative strengths for binary motion detection and four-class posture and motion recognition;
- A lightweight smart plug appliance recognition pipeline based on active power and power factor features is introduced for low complexity edge deployment to demonstrate the feasibility of integrating appliance-level behavioural sensing as a complemenatory branch for appliance-mediated IADL monitoring under low-complexity edge deployment constraints.

The remainder of this article is as follows. Related work in the field and the resulting knowledge gaps are discussed in Section 2. Given the findings of the literature review, the proposed methods are presented in Section 3, with the results and discussion reported in Section 4. Finally, this work is concluded and future work arising from the findings is suggested in Section 6.

## 2. Related Work

Ambient sensing modalities that can operate continuously within intelligent building environments are utilised to minimise user burden and privacy intrusion. Existing approaches span a broad range of sensing technologies, including mmWave radar, low-resolution thermal sensing, passive infrared sensing, WiFi-based sensing, and appliance-level power monitoring. Each modality captures different forms of behavioural evidence and exhibits distinct trade-offs in spatial resolution, motion sensitivity, deployment complexity, robustness, and privacy preservation. Multimodal architectures have emerged as a promising direction for ageing-in-place and ambient assisted living systems, as heterogeneous sensing modalities can compensate for each other’s characteristic limitations [16]. The ambient monitoring framework evaluated in this paper is therefore organised as a layered multimodal architecture consisting of (1) a foundation layer of 24-GHz consumer FMCW radar for whole-home presence, localisation, and motion sensing; (2) a middle layer of low-resolution thermal sensing for posture in high-interest zones; and (3) a per-plug power-monitoring layer for appliance-mediated IADL evidence. Each layer admits several competing sensing and modelling approaches, motivating the review presented in this section.

Numerous studies in ambient assisted living (AAL) have investigated ADL and IADL monitoring tasks, including mobility assessment, posture monitoring, kitchen appliance usage, hygiene routines, and fall detection. However, no single low-cost sensing modality adequately captures the full spectrum of behavioural evidence required for these applications, motivating the integration of multiple complementary sensing approaches [17,18,19,20]. Furthermore, the existing literature consistently highlights three critical unresolved challenges: selecting the optimal smallest possible sensor configuration to maintain adequate coverage of ADLs, preserving privacy in areas where social resistance is high, such as bedrooms and bathrooms, and guaranteeing fault tolerance in variable environments where network issues are common. A recent scoping review confirms the technological resistance in older adults’ acceptance of camera-based AAL, where non-visual ambient sensing was accepted in the same room [21]. The combination of requirements such as coverage, privacy, and fault tolerance frames the design space for the architecture proposed in this paper and motivates the layered, modality-diverse construction adopted below rather than a single-sensor or homogeneous modality alternative.

The foundation of a multi-modal AAL system requires whole-home coverage, robustness to occlusion, light, and darkness, stationary-occupant detection, and acceptance in bedrooms and bathrooms. While passive infrared radiometers (PIRs) offer low cost and minimal power consumption, their reliance on thermal gradient detection inherently fails to distinguish between a static occupant and the ambient environment. Utsumi et al. used retrospective algorithms to infer the missing gaps as a result [22]. Wi-Fi channel state information (CSI) is effective in controlled laboratory environments [23], but variability in consumer hardware and architectural layouts and the lack of native localisation capabilities results in reduced performance in real-world settings. Ultra-wideband (UWB) radar has been proven effective for fall detection, being robust with high accuracy and temporal resolution [24], yet these systems suffer from prohibitive deployment costs and regulatory restrictions. Consequently, mmWave radar sensors emerge as a critical modality occupying an optimal balance. These consumer-grade sensors (e.g., TI IWR1843 (Texas Instruments, TX, USA), Infineon BGT60 (Infineon Technologies AG, Neubiberg, Germany), and Hi-Link LD2450 (Shenzhen Hi-Link Electronic Co., Ltd., Shenzhen, China) offer a cheaper alternative, functioning effectively through some walls and in total darkness. Empirical studies further support the suitability of sensors in the lower frequency bands. Liang et al. [25] demonstrated that point-cloud enhancement combined with distribution-feature classification achieved fall detection performance exceeding 98.9% accuracy. The Radar-HAR literature has attempted multi-participant evaluation and the explicit study of generalisation. Gorji et al. [26] reported degraded accuracy once the subject or environment (unseen room layouts) deviates from the training distribution. They attempted to resolve lost generalisation using a two-stage classifier. Likewise, Li et al. [27] observed accuracy loses when applying deep learning models to new scenarios and used semi-supervised domain adaption to mitigate this. Crucially, this study employs the Hi-Link LD2450 mmWave radar due to its exceptional cost efficiency, making it among the most affordable sensors capable of generating robust track-level data (x, y, and velocity). This affordability, coupled with high refresh rates, ensures that the sensor provides a resource-efficient and foundational localisation layer for the multi-modal AAL architecture.

While the LD2450 radar sensor provides a robust foundational localisation layer, achieving whole-home coverage and reliable track-level data, it does not inherently provide the fine-grained posture recognition or highly specific fall detection required in critical, high-interest zones, such as over the bed or near bathroom doorways. This intermediate level requires adding dedicated functionality, specifically accurate posturing (lying, sitting, standing, or walking) in confined areas where occupant safety is paramount. Key activities such as falls and sleeping can be derived from posture by moving beyond detecting movement to defining the subject’s state and providing context for transitions [28]. For instance, differentiating between an occupant who slowly gets out of bed versus one who slips requires understanding the initial posture transition. Several modalities have been explored for this middle tier: depth cameras, multi-zone time-of-flight (ToF) sensors, acoustic sensing, and wearables. However, each approach faces significant technical or practical limitations when deployed in a real-world ambient assisted living (AAL) context. Depth cameras (Kinect or RealSense) (Microsoft, Redmond, WA, USA), (RealSense, Inc., Cupertino, CA, USA) offer rich 3D skeletal data and high accuracy in lab settings [29]. However, their clinical adoption is limited by the profound privacy concerns inherent to visual recording [21] and issues with hardware lifecycle management. ToF grids are highly attractive due to their affordability and privacy-preserving characteristics. At the time of this paper, commercially available options are restricted to severely low spatial resolutions, and their capacity for complex activity or posture detection remains sparse [30]. Acoustic systems utilise deep learning on audio signals [31]. While technically robust, they face strong practical rejection from older adults due to perceived surveillance (e.g., conversation recording). Low-resolution thermal arrays, such as the Melexis MLX90640 (Melexis Technologies NV, Limburg, Belgium) (32×24), Panasonic Grid-EYE AMG8833 (Panasonic Corporation, Tokyo, Japan) (8×8), and Heimann HTPA ( Heimann Sensor GmbH, Dresden, Germany), produce a silhouette-only image at 1–16 Hz at a unit cost comparable to a consumer PIR while categorically suppressing identity-relevant details. Newaz and Hanada [32] showed that posture-discriminative features remain present at low resolutions. Another study reviewed infrared sensing for human activity monitoring Muthukumar et al. [33].

The upper layer of human activity, such as meal preparation (stove or microwave), laundry cycles, or sustained desk work (computer), involves complex instrumental activities of daily living (IADLs). The current literature is dominated by two approaches: aggregate non-intrusive load monitoring (NILM) and per-appliance intrusive load monitoring (ILM). Research on electric load monitoring spans non-intrusive disaggregation from aggregate meters, plug-level intrusive monitoring, and edge-based implementations for real-time services. NILM has dominated the load monitoring literature, with most work focused on aggregated disaggregation from a single meter. However, a substantial gap remains between these disaggregation algorithms and deployable systems, particularly for real-world AAL scenarios and monitoring of ADLs [34]. Deep learning models maximise generalisation through long temporal contexts. Pujic et al. [35] employed a large window of 599 samples (around 30 min) in a domain adversarial neural network (DANN) to capture complete operational cycles. Similarly, transformer-based architectures often require large input windows to encode temporal dependencies effectively (e.g., 480 samples with a sampling rate of 6s [36]), rendering them unsuitable for immediate safety interventions. In these scenarios, ILM presents a more feasible path due to plug-level monitoring that provides physical isolation and localisation of current data directly from the appliance.

## 3. Materials and Methods

This section describes the experimental set-up (Section 3.1, dataset (Section 3.2), labelling methodology (Section 3.3), feature engineering (Section 3.4), model architecture (Section 3.5), and evaluation protocol (Section 3.6).

### 3.1. Experimental Set-Up

The workflow, shown in Figure 1, combines two separate complementary sensing streams as a redundancy-based resilience method: a radar–thermal HAR branch and a branch for the use of smart-plug appliances. The HAR branch processes radar and thermal data through labelling, windowing, feature extraction, classical and deep modelling, and fivefold grouped cross-validation using the macro F1 and related metrics. The smart plug branch processes 16 plug-level 1-Hz power streams through noise floor gating, multi-scale windowing, load-feature extraction, appliance modelling, and chronological train-validation–test evaluation. The output of the radar and thermal branches are integrated through fusion at the decision level to support low-cost redundancy, enabling robust monitoring of ADL and HAR when individual modalities are weak, ambiguous, or unavailable. The complementary system promotes continuous ADL and HAR recognition, resilient remote monitoring, intelligent buildings centred on the occupant, digital health services, and ageing well.

#### 3.1.1. Radar–Thermal Set-Up

**Figure 2:**
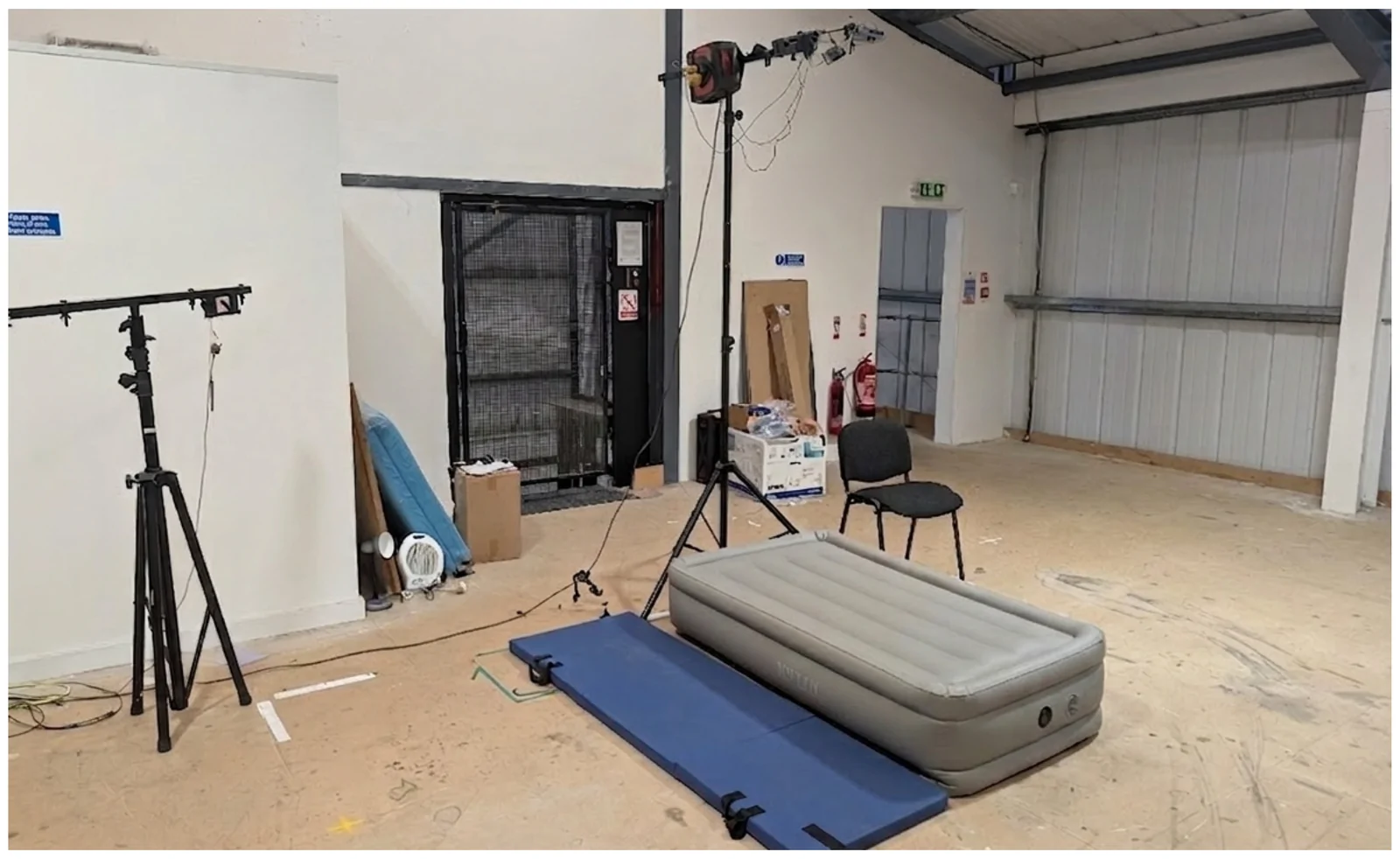
A photograph of the experimental set-up for E4, as described in Figure 3.

**Figure 3:**
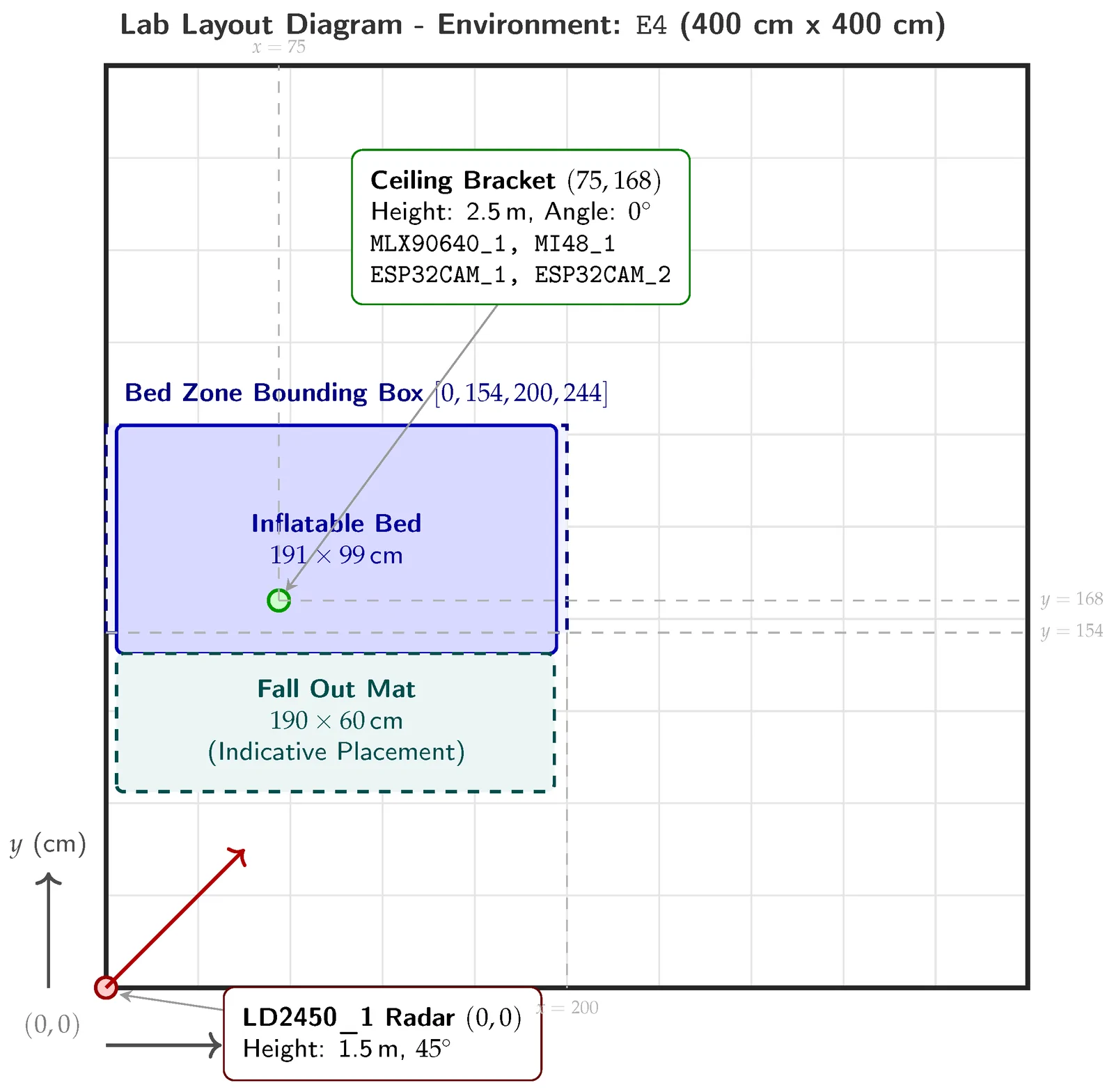
Diagram of experimental set-up E4 for data collection.

The experiments were conducted in a controlled indoor laboratory configured to emulate domestic activity scenarios shown in Figure 2. A Cartesian coordinate system was defined in a bird’s-eye view with the origin at the bottom-left corner of the room. The sensing platform consisted of one LD2450 mmWave radar sensor (Shenzhen Hi-Link Electronic Co., Ltd., Shenzhen, China), one MLX90640 thermal array (Melexis Technologies NV, Limburg, Belgium), one MI48 thermal imager (Meridian Innovation, Singapore), and two ceiling-mounted ESP32-CAM RGB cameras (Shenzhen Ai-Thinker Technology Co., Ltd, Shenzhen, China). The ESP32 cameras and the MI48 thermal imager were used as a reference. The radar sensor was mounted on the wall at 1.5 m with a $45^{\circ }$ orientation, while the remaining sensors were mounted at 2.5 m in a downward-facing configuration as seen in Figure 3.

Six room configurations were evaluated, varying the arrangement of furniture and environmental complexity, including empty room, bed-based, chair-based, fall mat, elevated bed, and dynamic layouts (Table 1).

Two ambient sensors were used for HAR. The Hi-Link LD2450 is a 24-GHz FMCW radar with on-board target detection and tracking. The LD2450 firmware exposes the planar position per target (x,y) in centimetres, the radial speed, and an event timestamp. Note that micro-Doppler signatures were not accessible and were therefore not used in this study.

With the LD2450’s $\pm 60^{\circ }$ horizontal aperture and ∼6m stated detection range, a single sensor at this mounting subtended the entire laboratory room used in the experiments. The Melexis MLX90640 is a 32×24 low-resolution thermal array with a configured refresh rate of 8Hz. It was ceiling-mounted at 2.5m and centred at a floor projection (x,y)=(75,168)cm from the room origin, being identical across all recordings. For purposes of labelling only, co-located on the same ceiling bracket were an MI48 thermopile array and an ESP32-CAM RGB module. These sensors were used to fine-tune the transition from one label to another.

#### 3.1.2. Intrusive Load Monitoring Set-Up

**Figure 4:**
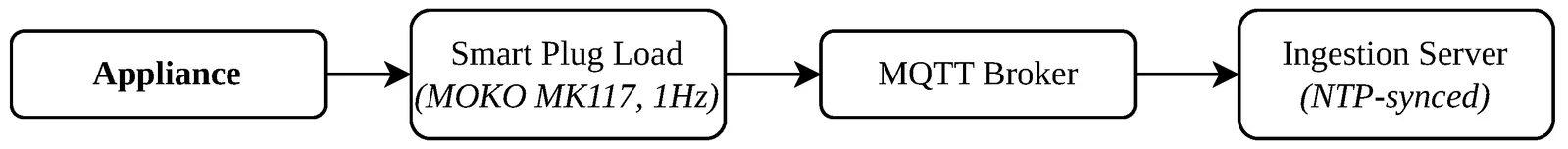
Smart plug acquisition pipeline for the appliance-load stream. Each plug publishes active power, power factor, current, and voltage to a local MQTT broker, while an NTP-synced ingestion server records the per-plug stream at 1Hz.

The third stream of the ambient stack instruments individual appliances with commodity smart plugs (MOKO MK117 (MOKO Technology Ltd., Shenzhen, China), equipped with an RN8209C power-metering chip (Shenzhen Renergy Technology Co., Ltd., Shenzhen, China). The plugs are run with their stock firmware, which supports local telemetry over MQTT with a quality of service level of 1 (at-least-once delivery) to prevent packet loss during transmission. Each plug reports the active power *P*, scalar power factor PF, RMS current *I*, and RMS voltage *V* at 1Hz. The pipeline is illustrated in Figure 4. Each appliance is wired through a plug; the plugs publish to a local MQTT broker over Wi-Fi; and an ingestion server, time-synchronized by NTP, consumes the broker stream and writes a long-form time series per plug to disk. The resulting per-plug dataset is referred to here as the Individual Load Monitoring (ILM) dataset to distinguish it from aggregated NILM corpora, such as UK-DALE, REFIT, and REDD, which sample at lower rates and do not expose a power factor channel [37,38,39].

Sixteen plugs were deployed on residential-grade appliances in an office setting, covering Type I resistive loads (kettle, toaster), Type II multi-state loads (microwave, fridge), and Type III continuously variable electronic loads (laptop, computer, and monitor), following the classification of Zoha et al. [40].

### 3.2. Dataset

Two datasets were used in this study. The HAR dataset contains paired LD2450 radar and MLX90640 thermal array recordings from a single subject across six furniture configurations in one room. Both sensing streams used identical window definitions, labels, and fold assignments, enabling direct comparison between radar and thermal baselines under matched evaluation conditions. The HAR data were evaluated using two label stages: a binary motion task and a four-class posture and motion task. The dataset composition, the windowing parameters, the class distributions, and the validation protocol for both HAR tasks are summarised in Table 2.

For the HAR experiments, cross-validation was performed using a fivefold recording-grouped protocol. Each sliding window inherited the recording identifier of the source recording from which it was extracted, and all windows from the same recording were assigned to the same fold. This prevented adjacent or overlapping windows from a recording appearing in both the training and held-out data, reducing leakage caused by temporal continuity and shared scene conditions. Stratification was applied during fold construction to preserve class balance as much as possible while treating the recordings, rather than individual windows, as the indivisible grouping units. The reported HAR results should therefore be interpreted as recording-held-out, within-subject estimates rather than cross-subject or cross-environment generalisation. The MI48, IMU, and RGB streams were recorded for labelling and context but not used as model inputs in the reported HAR classifiers.

The smart plug appliance-recognition branch uses a different data structure and validation strategy from the radar–thermal HAR tasks. The smart plug dataset contains per-plug power telemetry from 16 MOKO MK117 plugs attached to residential-grade appliances in an office setting. Each plug reports the active power, voltage, current, and scalar power factor at 1 Hz. The appliance recognition task was formulated as a five-class classification problem over appliance classes with at least three monitored instances, together with a consolidated Idle/Off class. The power-monitoring experiments used chronological train, validation, and test partitions with a 300-s safety gap between adjacent partitions to reduce leakage from overlapping windows. The smart plug dataset composition, feature representation, windowing parameters, and split protocol are summarised in Table 3.

### 3.3. Labelling

#### 3.3.1. Radar–Thermal

Each recording was labelled with SensorLogger [41], which emits class transitions as timestamped events. During data collection, several granular states, including “turning while lying down”, “lying back”, and “lying side”, were recorded and subsequently consolidated as needed. The “transition” label was removed from the dataset where there was not a clear distinction between classes. A preprocessing stage then expanded these events into a dense, per-frame label stream precisely synchronised with the radar and thermal timestamps. The final dataset comprised four distinct posture and motion classes: Lying, Sitting, Standing, and Walking. Two task stages were derived from the labels:

- **Binary motion (binary):** The labels were collapsed by mapping Walking to Moving and the three quasi-static classes, (Lying, Sitting, and Standing), to Stationary. The collapse yielded 8114 windows (2195 moving, 5919 stationary).
- **Four-class posture and motion (**four_class**).** The collapse yielded 7988 windows (2282 Lying, 1575 Sitting, 2062 Standing, 2069 Walking). The slight reduction relative to the binary stage was caused by stricter purity gating for the four-class task. A window whose label distribution was too mixed was discarded at the four-class stage but retained for the binary stage (see Section 3.4).

The four-class label set was chosen for three reasons. First, Lying, Sitting, Standing, and Walking were the four states that dominate the waking-hour budget of an elderly subject in a care-home room or a single-occupant home. Motivated by Katz et al. [42] and Lawton and Brody [43], these labels are routinely used as the operational vocabulary in clinical ADL and functional mobility assessments. The present single-subject data do not test this clinical interpretation. The walking versus non-walking distinction is the canonical cue for sustained physical activity. The sitting versus standing distinction matters for sedentary-time tracking. The lying class is essential for bed occupancy, sleep pattern, and prolonged recumbency monitoring in care home and ageing-in-place deployments. Second, this set is the smallest label vocabulary that simultaneously stresses the two behavioural axes identified in Section 1; the Walking class probes the motion axis on which the radar stream should be competitive, while the three quasi-static classes (Lying, Sitting, and Standing) probe the posture axis on which thermal sensing should hold the discriminative advantage. The four-class label set, therefore, lets a single evaluation simultaneously verify the binary motion finding within the broader posture context and quantify the posture decomposition advantage of thermal sensing within the stationary subset.

#### 3.3.2. Appliance Load Stream

Power factor readings were forced to unity when active power fell below a $\tau_{P}=5\;W$ noise floor threshold, and the corresponding sample was consolidated into a single idle class to prevent the model from learning spurious correlations from sensor jitter during off states. Minor gaps within active sessions were forward-filled. The thresholds were selected via manual inspection of the unlabelled power streams to ensure a robust separation between the hardware noise floor and each device’s active duty cycle.

### 3.4. Feature Engineering and Windowing

Each modality was processed by a per-stream feature engineering pipeline. The pipelines utilised constrained feature sets at constrained sampling so that any classical machine learning result on this dataset was a defensible deployment proxy for microcontroller or gateway-class hardware.

#### 3.4.1. Radar Features

Each radar window contained the sequence of LD2450 track samples falling within the window boundary. Two representations were produced:

- **Engineered-feature vector** for the classical models: For each window, summary statistics were computed over the per-target (x,y,v) stream, namely the central tendency descriptors (mean and median), dispersion descriptors (standard deviation and range), and motion-state descriptors derived from radial speed (mean speed, fraction of frames above a motion threshold, and longest stationary run inside the window). The track count and the in-window persistence of the dominant target were also included so that the classifier could distinguish a single occupant from intermittent track instability.
- **Sequence representation** for the deep models: The track stream was resampled and zero-padded into a fixed-length (T,3) tensor with T=20 steps per window, covering the canonical window cadence chosen for the LD2450.

#### 3.4.2. Thermal Features

Each thermal window contained a sequence of 32×24 frames from the MLX90640. Three representations were produced:

- **Engineered-feature vector** for the classical models: For each window, the per-frame summary statistics were aggregated over time. The per-frame statistics included the global pixel statistics (mean, standard deviation, minimum, maximum, and kurtosis), centroid coordinates of the above-threshold mass relative to the frame, projected silhouette area above an adaptive temperature threshold, and the dominant-blob aspect ratio. The threshold was derived per-window from the in-window background temperature distribution so that occupant-versus-environment separation was not tied to absolute calibration. The aggregated time series statistics captured posture stability (variance of centroid position over the window) and motion presence (centroid trajectory length).
- **Single representative frame** for the static deep models, taken as the central frame of the window for the capacity-matched 2-D CNN arms.
- **Frame sequence** for the temporal deep models: A T×32×24 tensor was implemented where *T* matched the radar window cadence so that the two modalities shared a common per-window time base under late fusion.

#### 3.4.3. Appliance-Load Features

Each smart plug window of a duration *W* (with W∈{10,60,300}s in the ablation study) was summarised by a 16-dimensional feature vector $x\in R^{16}$ built from the central-tendency, dispersion, and shape statistics, namely the mean, standard deviation, minimum, maximum, skewness, kurtosis, and range, extracted from both the active power (P) and power factor (PF) channels. The feature space was restricted to statistical descriptors of *P* and PF so that the entire computation was a small fixed-point reduction over the window with no FFT or large state buffer, which is feasible on microcontroller-class hardware. The same 16-dimensional vector is the input to all three appliance models (Section 3.5.2). The $\tau_{P}=5\;W$ noise floor gating and the Idle consolidation defined in Section 3.3.2 were applied upstream of feature extraction.

### 3.5. Model Architecture

#### 3.5.1. Radar–Thermal Models

For HAR, the classical machine learning, deep learning, and temporal deep learning approaches were benchmarked as follows:

**Radar classical baselines:** Four classical learners were trained on the handcrafted radar features, namely logistic regression, random forest, multilayer perceptron (MLP), and XGBoost [44]. The tunable hyperparameters were the regularisation strength for logistic regression, the number of trees and maximum depth for random forest and XGBoost, and the hidden-layer sizes for the MLP.

**Radar deep learning:** Five deep approaches were trained on the (T=20,3) radar track sequence, specifically an MLP on handcrafted features and, for the raw data, a 1-D CNN, LSTM, CNN-LSTM, and a transformer [45]. The 1-D CNN grid covered channel widths {16,32} and had a hidden depth of {2,3}. The LSTM grid covered the hidden sizes {32,64} of layer counts {1,2}. The CNN-LSTM grid covered CNN channel widths {16,32} and LSTM hidden sizes {32,64}. The transformer grid spanned $d_{model}\in \left\{16,32,64\right\}$, encoder depths {1,2}, attention heads {1,2,4}, dropout of {0.1,0.3}, and learning rates of $\left\{10^{-3},3\times 10^{-4}\right\}$.

**Thermal classical baselines:** As with the radar classical baselines, the same four classical learners (logistic regression, random forest, MLP, and XGBoost) were trained on the handcrafted thermal features extracted per window.

**Thermal static deep learning:** Three 2D CNNs were trained on a single representative 32×24 frame per window. Model sizes are given as *small* (depth = 2, channels {16,32}), *mid* (depth = 3, channels {16,32}), and *deep* (depth = 4, channels {16,32}). An MLP on the flattened frame provided the baseline. All models tuned the learning rate $\left\{10^{-3},3\times 10^{-4}\right\}$.

**Thermal temporal deep learning:** Four temporal models were trained on the full T×32×24 frame sequence per window, namely a 3D CNN with two channel configurations ((8,16) and (16,32)), a small ConvLSTM with 16 CNN channels and ConvLSTM hidden sizes of {16,32}, a frozen-2D CNN backbone followed by mean pooling over time, and a CNN with a temporal pooling head over channel widths of {16,32} and pool types {mean,max}.

**Neural training protocol:** All neural arms used AdamW, with a weight decay of $10^{-4}$, class-weighted cross-entropy with label smoothing of 0.05, dropout defined by the architecture grid, gradient clipping at 1.0, early stopping, and three-seed refits of the selected configuration. Radar and static thermal neural arms used a batch size of 64, at most 60 epochs, patience of 8, and a 120-min inner-search budget. Temporal thermal arms used a batch size of 32, at most 50 epochs, patience of 6, and a 180-min search budget because the frame sequences had a larger memory footprint. These configurations represent systematically explored baselines under the grouped-CV protocol and not exhaustively optimised designs.

#### 3.5.2. Appliance Recognition Models

The appliance-recognition pipeline aims to learn per-plug classification of the appliance type from a window of telemetry. The 16-dimensional feature vector $x\in R^{16}$ over the (P,PF) channels is defined in Section 3.4.3. Three model families were benchmarked under matched random seeds on this vector: a random forest (100 trees, no maximum depth), an XGBoost classifier (100 trees, maximum depth of 6, learning rate of 0.3, histogram tree method), and a compact two-layer MLP with hidden sizes of (64,32), ReLU activations, and approximately 3.5k parameters chosen to remain feasible on resource-constrained edge gateways, such as the Raspberry Pi Zero. The empirical comparison of these arms is reported in Section 4.5.

### 3.6. Evaluation Protocol

All HAR comparisons reported in this paper used a single stratified fivefold partition grouped by recording identifier. Grouping by recording was the leakage-controlling substitute for participant or environment grouping in this single-subject, fixed-placement dataset. The resulting evidence therefore supports within-subject, within-placement comparison only and does not estimate the transfer to new participants, sites, or sensor placements.

Per fold, the training portion was used for inner hyperparameter searching under a per-arm time budget. The inner search used a held-out validation slice grouped by the same recording identifier. The selected hyperparameters were then refit on the full training portion across multiple seeds and applied to the held-out test fold. The outer metrics were aggregated across the five test folds. No information from the outer test fold entered hyperparameter selection, feature-scaling statistics, or early-stopping decisions. The neural arms additionally averaged three seed refits of the selected configuration, and thus the reported metrics were not contingent on a single initialisation.

For HAR comparisons, the reported metric set was as follows: the accuracy, balanced accuracy, macro $F_{1}$ (the headline metric for all winner selection), one-vs.-rest macro-AUC, and per-class precision, recall, and $F_{1}$. Confusion matrices are reported per task per modality from the pooled held-out predictions across all five folds. Here, pooled held-out predictions means that the test predictions from all five outer folds were concatenated after each sample was evaluated only by the model that did not train on it. Numerical fold-level metrics are reported as the mean ± standard deviation over the five folds.

Reproducibility of the reported comparisons is supported by a frozen fold definition, fixed preprocessing rules, fixed hyperparameter budgets, and recorded random seeds for each evaluated arm.

### 3.7. Appliance-Load Evaluation

The appliance-load benchmark used chronological partitioning into training (70%), validation (15%), and test (15%) sets, with an enforced temporal safety gap between adjacent partitions equal to the maximum window duration ($W_{\max}=300\;s$) to prevent look-ahead bias from sliding-window segmentation. The reported metric is the macro-averaged $F_{1}$ over appliance classes, with paired statistical comparisons via the Wilcoxon signed-rank test for feature-set ablations. The natural integration route for the three-stream stack was to add per-plug appliance-classifier outputs as additional inputs to the late fusion combiner already used for the radar–thermal pair in Section 4.4.

## 4. Results

This section reports the HAR results on the binary motion and four-class posture and motion tasks, followed by the smart plug appliance recognition results. Section 4.1 and Section 4.2 report the HAR per-modality baselines. Section 4.5 reports the appliance-load branch. Section 4.3 pairs the two modalities on the same folds with a paired Wilcoxon signed-rank test. Section 4.4 reports the late-fusion.

**Figure 5:**
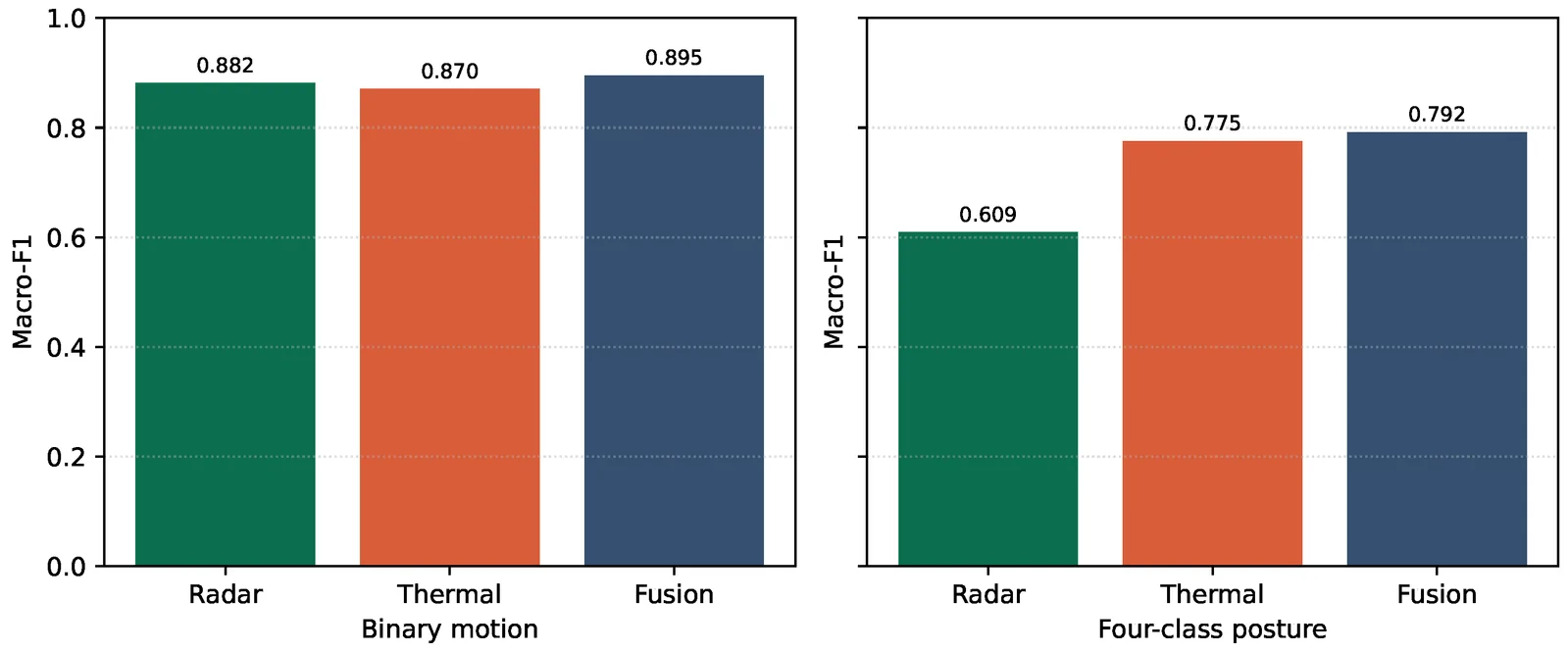
Task-level macro $F_{1}$ summary for the radar winner, the thermal winner, and the best late-fusion pair from the matched comparison.

Table 4 consolidates the full metric set for the best HAR unimodal pipeline on each task, including the balanced accuracy and macro-AUC. Figure 5 places the two unimodal winners and the late-fusion result on the same scale. Radar and thermal were nearly indistinguishable on binary motion, whereas the four-class task was dominated by thermal and then lifted further by late fusion.

### 4.1. Binary Motion Overview

Table 5 reports the radar classical sweep. Random forest was the best radar classical arm on both tasks, reaching a 0.853±0.082 macro $F_{1}$ on binary motion and 0.609±0.110 on the four-class task.

Table 6 reports the radar deep learning sweep. The transformer was the best binary arm at a 0.882±0.034 macro $F_{1}$, above the radar ML binary winner by 0.029 in terms of macro $F_{1}$. On the four-class task, the best deep arm was the feature MLP at 0.567±0.077, below the radar classical four-class winner. Radar sequences are useful for gross motion, but the engineered radar features remained stronger for the four-class task.

**Figure 6:**
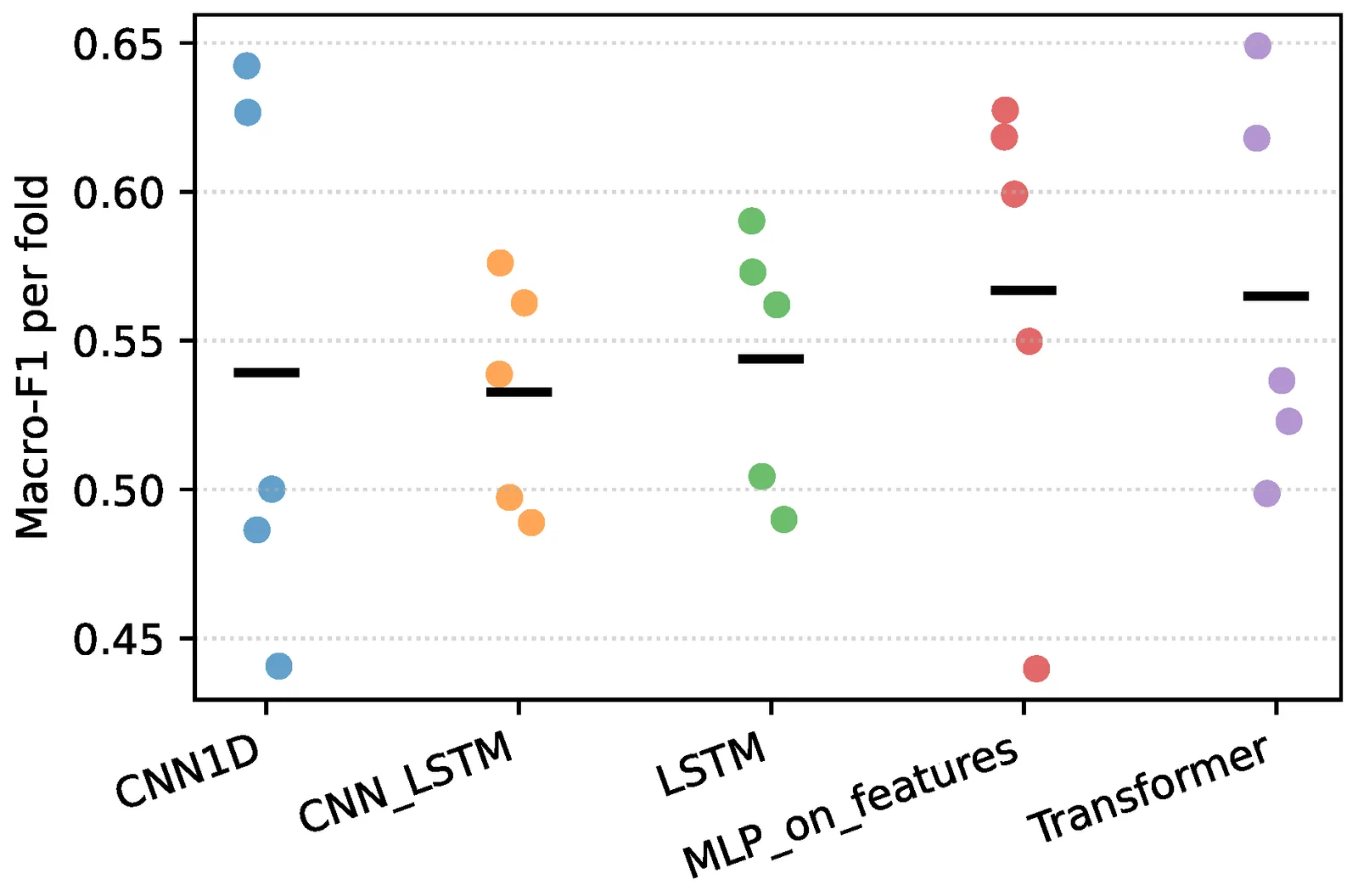
Radar deep family per-class macro $F_{1}$.

Figure 6 shows the per-fold macro $F_{1}$ spread for the radar deep family on the four-class task. The fold-wise spread was wide for the sequence-oriented arms, consistent with the limited spatial detail in the track representation.

### 4.2. Thermal Baselines

Table 7 reports the thermal classical sweep. XGBoost was the best thermal classical arm on binary motion (0.870±0.069 macro $F_{1}$). The random forest model was the best four-class arm (0.775±0.053). The four-class result was the strongest completed multi-class result among the final thermal runs and aligned with the expectation that low-resolution thermal arrays carry posture and silhouette information that sparse radar tracks may miss.

Table 8 and Table 9 show the static 2D CNN family peaks in the mid configuration on both tasks (0.761±0.070 on binary and 0.608±0.120 on four-class) and the temporal family peaks at ConvLSTM-small on both tasks (0.839±0.061 on binary and 0.732±0.079 on four-class). Temporal modelling was therefore the stronger thermal deep learning regime, but the best thermal classical arm remained stronger on four-class than either thermal deep family. This is consistent with the argument that thermal arrays preserve enough spatial structure for posture, while temporal context helps most when the class boundary depends on motion over time.

**Figure 7:**
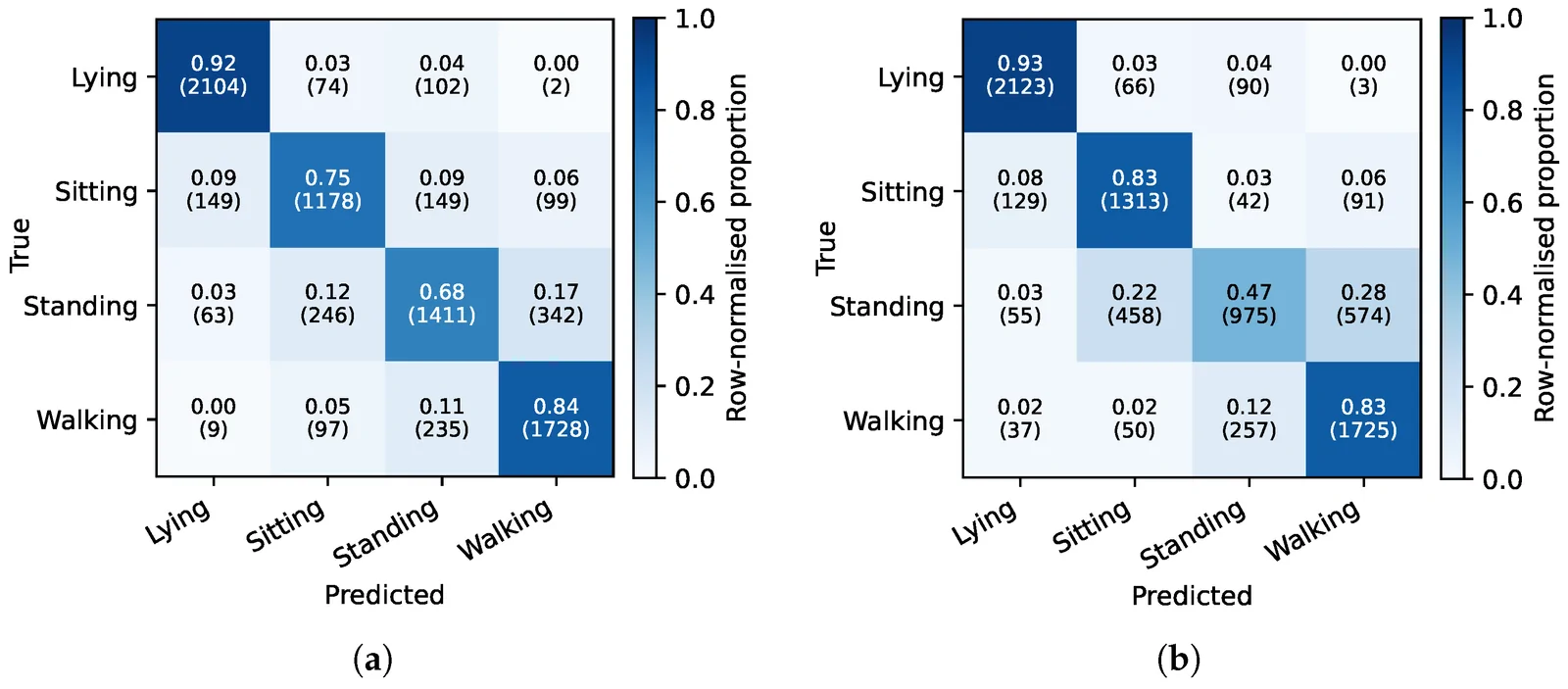
Row-normalised confusion matrices for thermal four-class classification aggregated across five recording-grouped folds. (**a**) Thermal ML, four-class pooled confusion matrix. (**b**) Thermal temporal-deep model, four-class pooled confusion matrix.

Figure 7 shows the pooled held-out confusion matrices for the best thermal classical arm and the best thermal temporal deep arm on the four-class task. Both matrices showed strong recognition of lying and walking, but their failure modes differed. The random forest preserved standing more reliably, whereas ConvLSTM-small improves sitting recall at the cost of increased standing confusion, particularly with walking and sitting. Temporal modelling was useful within the thermal deep family, but the classical random forest remained the stronger four-class thermal arm.

### 4.3. Modality Comparison

The two modalities were compared on the same five folds using fixed arms selected from the unimodal evaluations. Table 10 reports the pairing. The comparison resolved into two distinct regimes. On binary motion, the two modalities were within fold-wise noise of each other (0.882±0.034 radar versus 0.870±0.069 thermal), and thus the low-cost radar stream was competitive for the coarse motion-gating role. On the four-class task, thermal was ahead of radar by 0.166 in terms of macro $F_{1}$ (0.775±0.053 versus 0.609±0.110), which is the first direct indication that redundancy matters, because the two streams were no longer interchangeable once posture entered the label space.

The fold-paired Wilcoxon tests showed different modality comparison patterns across the two tasks. On binary motion, the test did not reject the null hypothesis (the difference was not statistically resolved at the available fold count). On the four-class task, the fold-paired *p* value remained borderline at k=5, even though the thermal advantage was consistent in magnitude and direction. Radar carried the motion signal at parity with thermal, which supports the Tier 1 radar-only deployment rationale referenced in Section 1. Thermal carried the posture signal that radar at this representation level did not, which is exactly what is required to motivate adding the thermal stream at Tier 2.

**Figure 8:**
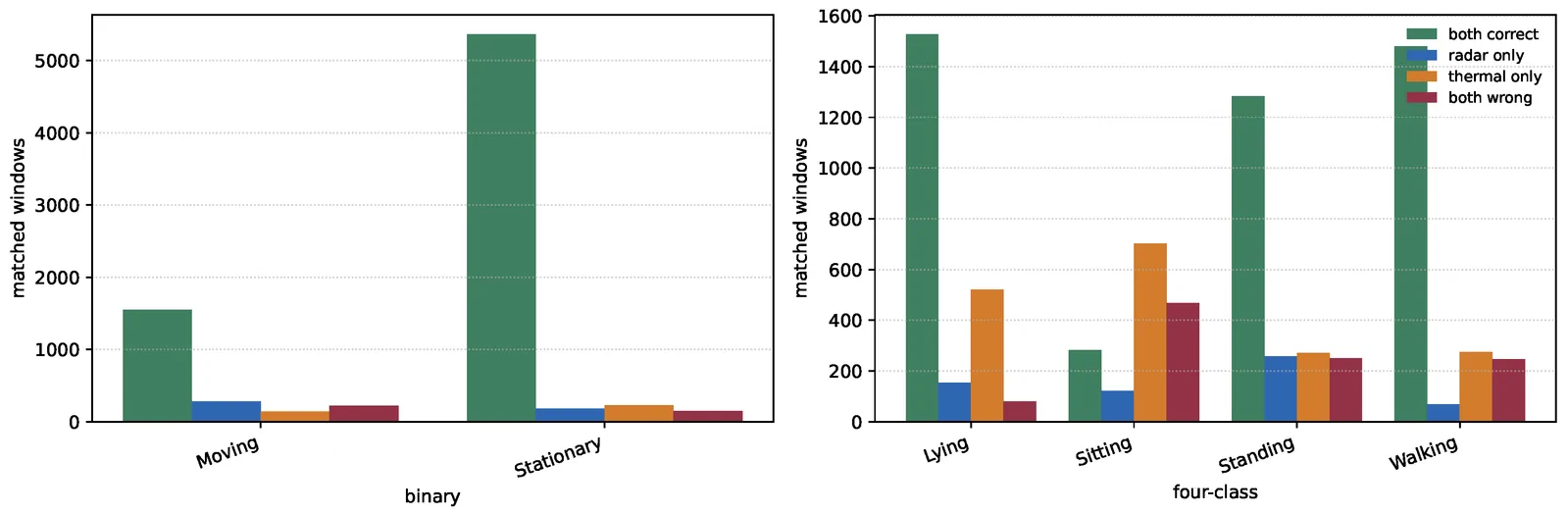
Per-class matched-window winner matrix for the fixed radar and thermal arms.

**Figure 9:**
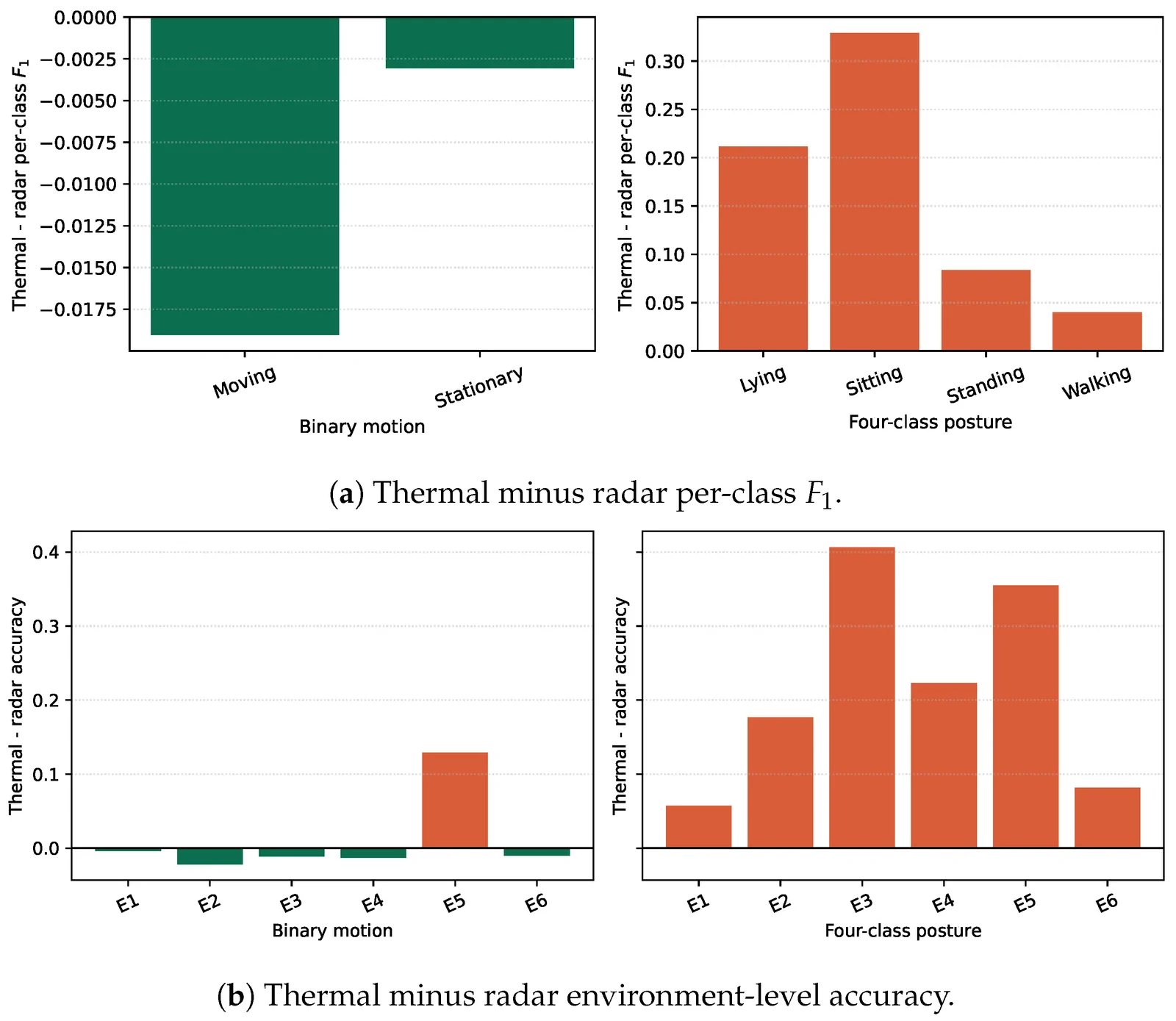
Binary motion showed only small class and environment-level differences, whereas the four-class task showed a consistent thermal advantage driven mainly by posture-sensitive classes.

Figure 8 and Figure 9a unpack this aggregate comparison. In the binary task, radar had more radar-only correct windows for Moving, while the two modalities were close on Stationary. In the four-class task, the largest thermal-only counts occurred for Lying and Sitting, and the per-class macro $F_{1}$ gaps were largest for Sitting (+0.329) and Lying (+0.211). The smaller gaps for Standing (+0.084) and Walking (+0.040) support the interpretation that the modality gap is mainly a posture decomposition effect rather than a coarse motion effect.

Figure 9b decomposes the same comparison by class and by environment. On binary motion, the class-wise differences were negligible, and most environments favoured radar only slightly. The two modalities were practically tied at this label granularity. On the four-class task, the thermal advantage was concentrated in Sitting and Lying, while Walking was nearly tied, precisely the per-class pattern that the radar’s lack of posture detail predicted and the thermal sensor’s silhouette information delivered. The environment-level view was consistent with the class-level view that thermal was ahead in every four-class environment, with the largest gains in the environments that contained the strongest posture variation (bed and chair-plus-bed configurations).

**Figure 10:**
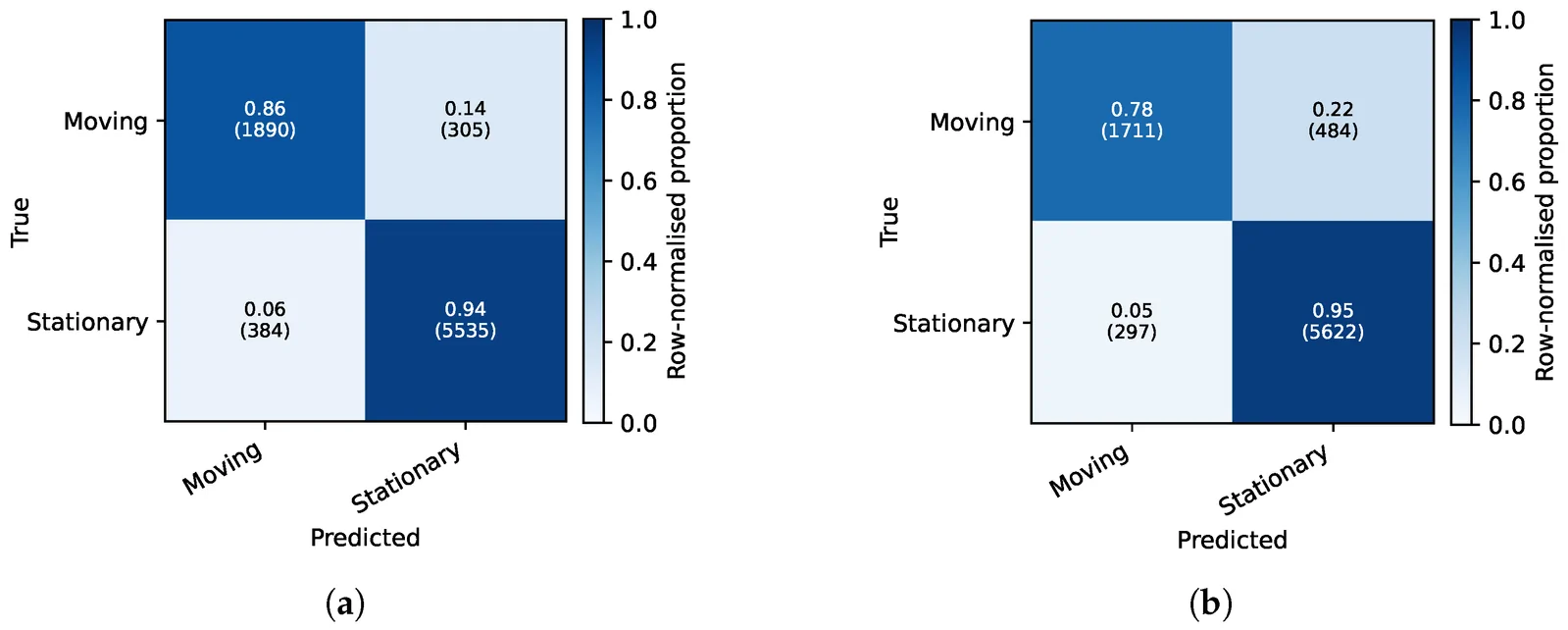
Pooled held-out confusion matrices for the binary motion task, row-normalised, across the five recording-grouped folds. (**a**) Radar winner (transformer), binary task, row-normalised pooled confusion matrix. (**b**) Thermal winner (XGBoost), binary task, row-normalised pooled confusion matrix.

Figure 10 shows the pooled held-out confusion matrices for the best binary winner on each modality. Both matrices were diagonal-dominant. The residual error was concentrated on the moving class, consistent with the class imbalance (27% moving) and with brief stationary-moving transitions at activity boundaries. Both sensors made essentially the same kinds of mistakes on the motion axis. Because the monitoring rationale rested on detecting movement and motion onset, the minority Moving class (27% of the binary windows) was the operationally costly error direction. A moving window misclassified as stationary is a missed event, whereas the reverse error is comparatively benign. The binary confusion matrices (Figure 10) concentrated the residual error on exactly this class, and thus the headline macro $F_{1}$ understated the cost asymmetry. A deployment would bias the operating threshold towards higher Moving recall and accept lower precision.

### 4.4. Fusion Status

Late fusion combines fixed-arm per-modality probability outputs on the same five recording-grouped folds using a scalar radar–thermal mixing weight w∈[0,1] that forms the fused posterior as $w\;p_{\mathrm{radar}}+\left(1-w\right)\;p_{\mathrm{thermal}}$. For each outer-training fold, the weight was swept over a grid spanning [0,1] at a step of 0.1 and selected to maximise the macro $F_{1}$ for inner-CV out-of-fold predictions drawn only from that outer-training split. The selected weight was then applied unchanged to the held-out outer-test fold.

Table 11 reports the single-modality references and the distinct fused candidates. Pair A denotes the classical machine learning baseline pair, whereas Pair B denotes the pair formed from the unimodal winners. For the four-class posture, both definitions used the radar random forest and thermal random forest, and thus the four-class analysis contained one distinct fused candidate rather than two independent pair definitions.

On binary motion, the best fused arm reached a macro $F_{1}$ of 0.895±0.039, compared with 0.882±0.034 for the radar transformer and 0.870±0.069 for thermal XGBoost. On the four-class task, the fused arm reached 0.792±0.051, compared with 0.775±0.053 for the thermal random forest and 0.609±0.110 for the radar random forest. These gains are compatible with complementary radar–thermal failure modes, but the five-fold paired tests did not establish a statistically resolved improvement over the stronger unimodal reference (p=0.4375 for binary motion and p=0.1875 for four-class posture).

**Figure 11:**
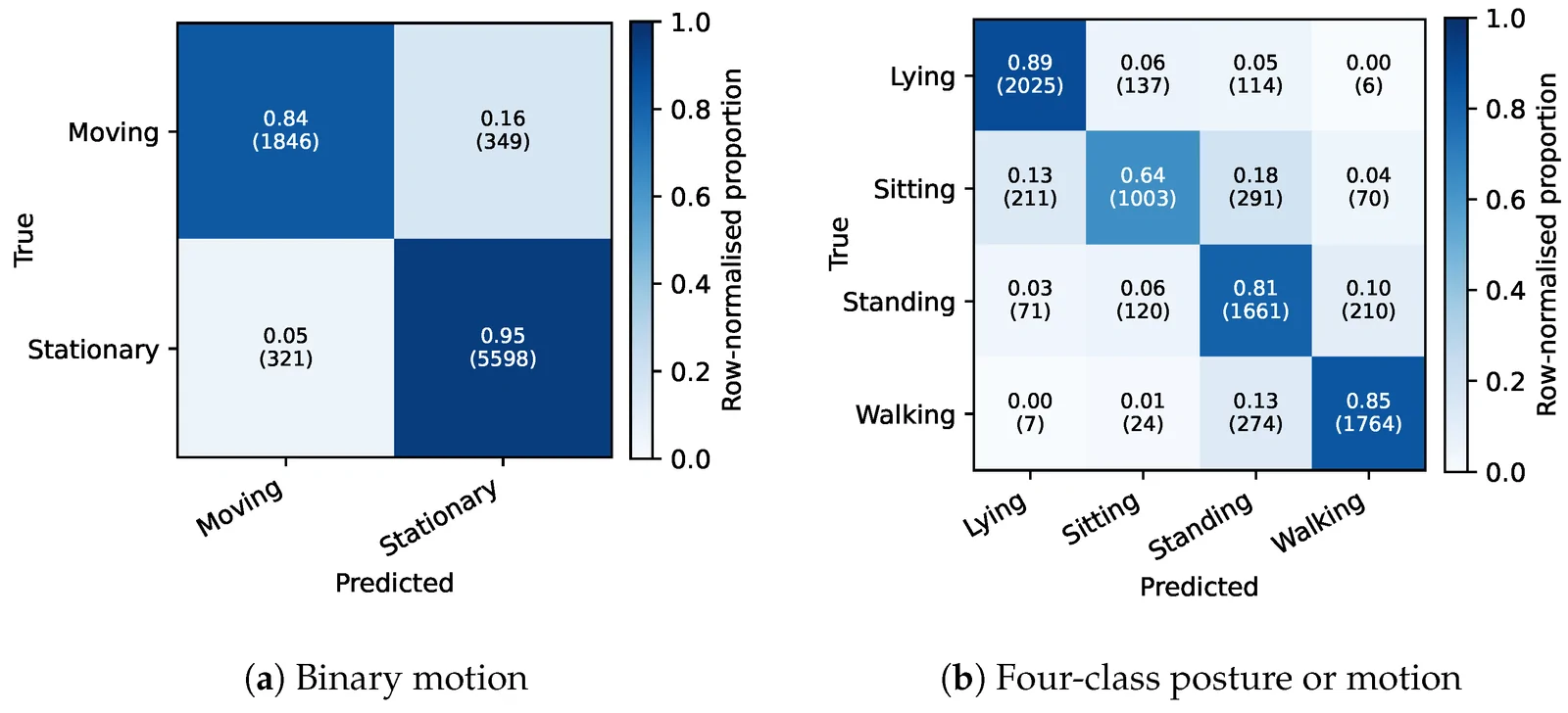
Late fusion confusion matrices for the binary motion and four-class posture and motion tasks.

Figure 11 shows the confusion matrices of the best fused system on both tasks. On binary motion, the fused classifier retained the strong stationary-class performance of the unimodal models while reducing moving-class misses. On the four-class task, the fused classifier improved all four classes relative to the radar winner and most clearly sharpened the Sitting and Lying boundaries that drove the unimodal modality gap.

**Figure 12:**
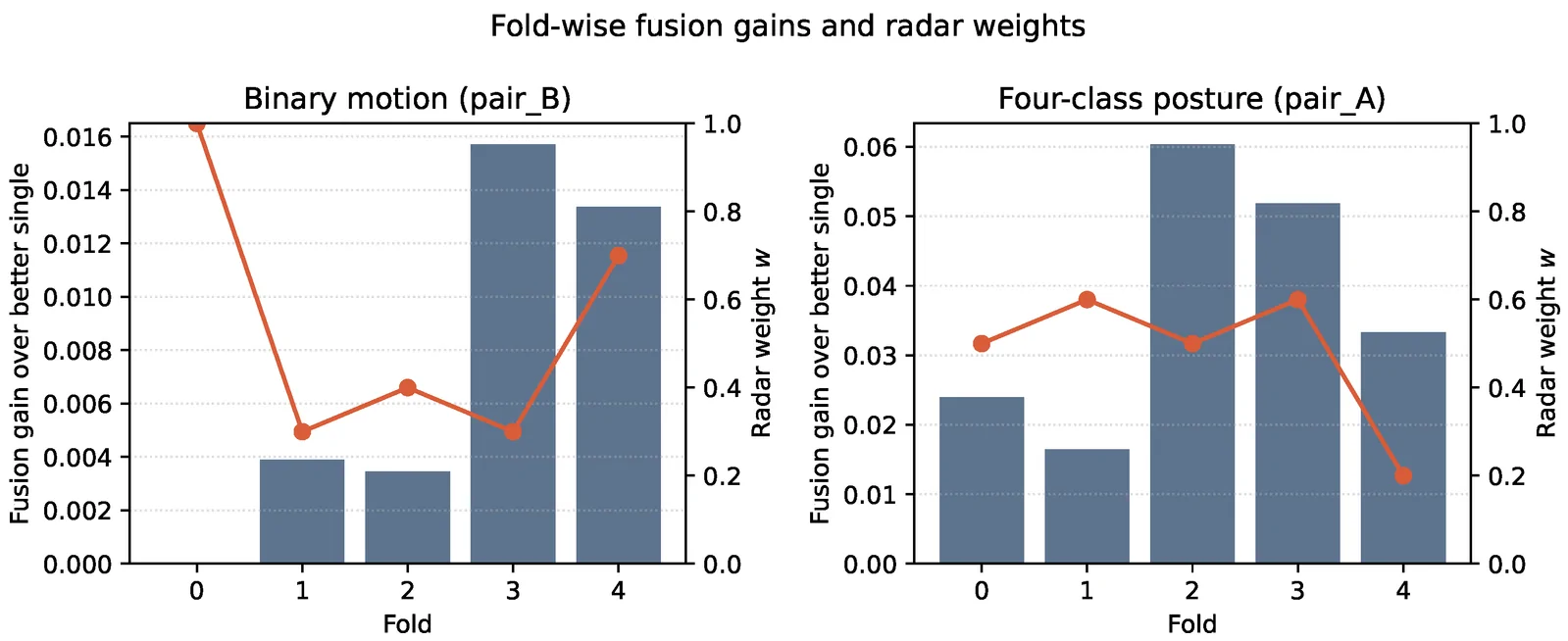
Fold-wise fusion gain over the better unimodal reference and the corresponding radar weight of the best fused pair on each task.

Figure 12 shows how the fusion gain was distributed across folds together with the learned radar weight. The binary gain was small and fold-dependent. One fold collapsed to radar-only weighting (because the binary radar arm was already saturating performance for that fold’s environment mix), and the remaining folds gained at most 0.016 for the macro $F_{1}$ over the better single modality. By contrast, the four-class gains were positive on all five folds (0.016–0.060 macro $F_{1}$) with mixed radar weights between 0.2 and 0.6, consistent with genuine complementarity rather than one modality dominating the combiner.

### 4.5. Smart Plug Appliance Recognition

The smart plug branch was evaluated as an appliance recognition task on windows of active power and the scalar power factor. Table 12 summarises the best 10-s window configuration for each model family. The two tree ensembles were statistically close in terms of macro $F_{1}$, with random forest reaching 0.977±0.002 and XGBoost reaching 0.976±0.004. The practical deployment trade-off favoured XGBoost. It retained the same accuracy regime while reducing the serialised footprint from 1.5 MB to 0.828 MB and reducing the measured inference latency from 38 ms to 0.4 ms. The compact two-layer MLP had the smallest footprint but was not competitive in terms of macro $F_{1}$.

**Figure 13:**
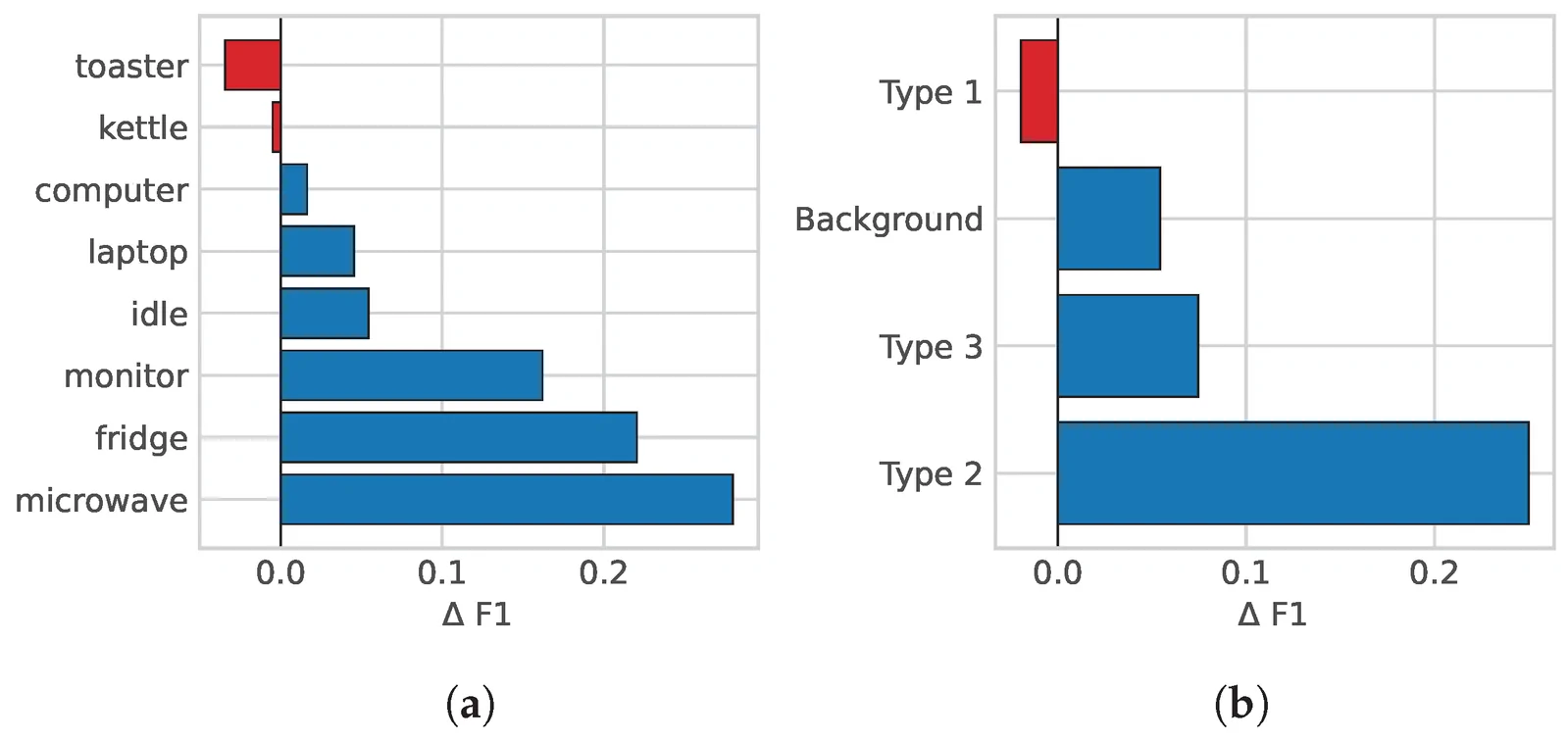
Power factor feature ablation. Each bar shows δF1 = F1 (with power factor channel) − F1 (without power factor channel), and thus positive values indicate that including the power factor channel improved classification. Panel (**a**) breaks the effect down per appliance, and panel (**b**) aggregates per appliance type. Red indicates reduced performance.

The appliance benchmark peaked at short windows. The 10-s setting gave the best macro $F_{1}$, the 60-s setting remained usable at roughly 0.929, and the 300-s setting dropped to about 0.74 as short duty cycles were diluted inside longer windows. Figure 13 shows the power factor ablation that explains why active power alone is not enough for several appliance classes.

The appliance result extended the activity space rather than competing with the radar and thermal HAR classifiers. While the radar and thermal sensing observe body motion and posture, the smart plug branch observes the instrumented appliance state associated with the instrumental ADLs explicitly catalogued by Lawton and Brody [43], such as cooking, kettle use, microwave use, and sustained desk activity. Once mapped onto that IADL vocabulary, the branch supplied evidence for the appliance-mediated half of the framework that the body-sensing streams could not recover: did the occupant make tea today (kettle), use the microwave (Type II appliance), or simply remain at the desk (computer and monitor active for a sustained window)?

## 5. Discussion

### 5.1. Sensor Roles and the Value of Each Tier

On binary motion, the two modalities were within 0.011 of each other in terms of macro $F_{1}$ in the matched comparison (0.882 radar transformer vs. 0.870 thermal XGBoost), and the paired Wilcoxon test did not reject the null hypothesis. The sparse-track (x,y,v) representation is therefore sufficient for the role assigned to the radar stream in Section 1 at a fraction of the hardware and bandwidth cost. This parity is the empirical foundation of the Tier 1 deployment. A care home room can be equipped with a single LD2450-class radar with a binary motion classifier running on a microcontroller or gateway-class endpoint and still recover the in-bed or out-of-bed signal that supports presence-based and fall-adjacent monitoring. The radar’s wide field of view and robustness to ordinary furniture occlusion give it an additional deployment edge for this tier that the thermal sensor does not match. A single LD2450 covers a complete room from a wall mount, whereas the ceiling-mounted MLX90640 only sees the slice of the room directly below it.

For the four-class results, the thermal random forest reached 0.775, the radar random forest reached 0.609, and the fold-paired comparison remained directional but underpowered at k=5. The gap of 0.166 for the macro $F_{1}$ was concentrated in the three quasi-static classes; thermal’s Walking performance was within 0.040 of radar’s while Sitting (+0.329) and Lying (+0.211) together accounted for roughly 82% of the gap, and Standing (+0.084) contributed the remainder (Figure 9a). This matches what the representation predicts: the track level (x,y,v) collapses to a near-zero-speed cluster across the three stationary classes, and posture decomposition within that cluster requires spatial detail the radar firmware discards before output. If the deployment needs to distinguish postures such as fall versus sitting on a bed, lying versus sitting in a chair, or prolonged recumbency versus active rest, then the thermal stream is the load-bearing modality, and adding it is justified. The thermal confusion matrices also explain why the engineered-feature random forest remained competitive against temporal thermal deep learning. Lying was recognised reliably by both thermal families, consistent with its distinct spatial footprint in the overhead low-resolution array. ConvLSTM-small improved sitting recall but lost standing recall, with standing more often being confused with walking or sitting. This suggests that short-term dynamics help stable seated postures but are less reliable for separating upright static posture from motion-adjacent windows in this dataset. Engineered thermal summaries appear to preserve posture geometry more effectively under the present single-subject, fixed-placement protocol.

### 5.2. Deep Learning on Track-Level Radar

The radar deep arms beat the radar classical baseline on binary motion but not on the four-class task. This is consistent with the data budget and with the representation where deep sequence models gain little when the input is three scalar streams per track. The radar deep learning result should therefore be read as a baseline for the sparse-track representation and not as a general claim about radar deep learning.

### 5.3. Late Fusion

Late fusion improved on the better unimodal pipeline on both tasks, but the operational reading was asymmetric. On binary motion, the gain was modest. For a deployment that only needs binary motion, the added sensor cost of running both modalities is not justified by the fusion gain alone. The Tier 1 radar-only stack remained the right design choice. On the four-class task, all five folds experienced gains with mixed radar weights, which is the signature of genuine modality complementarity rather than one modality dominating the combiner.

### 5.4. The Smart Plug Branch for IADLs

The smart plug result added a different kind of evidence from the radar–thermal HAR results. Its task was not posture recognition but appliance-state recognition, which mapped onto the instrumental-ADL axis that body-sensing streams cannot observe directly. The high macro $F_{1}$ values for random forest and XGBoost are credible within that narrower setting because the plugs isolate individual appliance loads before classification. XGBoost retained the random forest accuracy regime while reducing both the serialised size and inference latency, making it the better candidate for an edge gateway.

The power factor ablation is the strongest evidence that this branch is not simply learning appliance wattage. Scalar PF adds information for electronic and inductive loads whose active power statistics overlap, and its removal lowered the macro $F_{1}$ and increased variance in the ablation grid. This supports the design choice to use commodity plugs that expose more than active power alone. The limitation is equally clear that the result is bounded to known appliance classes in one office dataset. It does not establish transfer to unseen homes, unseen device instances, or activities without instrumented appliances.

### 5.5. HAR Applications

The results suggest that relying on a single sensor for privacy-preserving human activity recognition (HAR) may be insufficient when both motion sensitivity and posture awareness are required. Instead, the data strongly advocate for a layered sensing architecture where radar, low-resolution thermal imaging, and smart plug power monitoring are hierarchically integrated based on their distinct strengths and weaknesses. Radar yielded whole room coverage and reliable binary motion mapping but failed at posture detection. Low-resolution thermal sensing successfully captured both, but its narrow field of view (FoV) and susceptibility to occlusions make whole-room deployment impractical. By treating these modalities as complementary rather than competitive, a clear hierarchy emerges. Radar serves as the continuous spatial baseline, while thermal sensors function as a targeted secondary layer deployed strictly in high-risk or high-interest zones (e.g., beds or chairs) where postural context is critical.

While radar and thermal sensors map physical mechanics, they lack semantic clarity. Conversely, plug-level power monitoring captures specific activities of daily living (ADLs), though such data are often temporally sparse (e.g., turning on a kettle and walking away). The system’s appliance classification model resolves this by dynamically identifying devices via unique load signatures, extracting immediate activity context, and establishing localized spatial anchors at the moment of activation. Eliminating hardcoded socket-to-appliance mappings ensures these smart plugs remain fully interchangeable and scalable. Within this hierarchical architecture, the resulting inter-layer redundancy serves as a deliberate robustness mechanism rather than an inefficiency. Specifically, thermal arrays provide localised motion backup during radar signal degradation; appliance activations provide high-accuracy spatial anchors to bound continuous radar tracking drift; and both modalities dual-verify high-consequence events like falls. Because these overlapping capabilities possess distinct, known constraints, they offer complementary protection against single-modality failures without compounding data overhead.

### 5.6. Limitations and Scope of Generalisation

These findings should be interpreted as a controlled initial validation rather than evidence of deployment-level generalisation. The HAR experiments use a single subject, fixed sensor placements, and one room with varied furniture configurations. As a result, generalisation to different participants, body sizes, gait patterns, clothing, thermal emissivity conditions, room geometries, mounting heights, and sensor orientations were not estimated.

Prior radar-HAR work has shown that cross-subject transfer can produce substantial performance degradation [26]. Therefore, a larger multi-participant study with leave-one-subject-out and leave-one-environment-out evaluation is required before making broader deployment claims.

The validation protocol also has a bounded scope. Recording-grouped cross-validation reduces adjacent-window leakage, but it does not replace subject-independent or environment-independent testing. Similarly, the radar results apply specifically to the sparse LD2450 track representation used in this study and should not be interpreted as a general limitation of mmWave radar. Richer radar representations, such as point clouds, range–Doppler maps, range–angle maps, or micro-Doppler signatures, may provide additional discriminatory information and should be compared in future work.

The fusion and deployment analysis are likewise limited. Radar–thermal fusion was evaluated only through late decision-level fusion, motivated by the different sampling rates and feature spaces of the two modalities. Early, intermediate, temporal, and uncertainty-aware fusion strategies were not benchmarked. In addition, although the selected sensors and model families are compatible with constrained edge deployment, this study did not include a full target-device audit of latency, throughput, power draw, memory use, or long-run stability.

Finally, the proposed radar–thermal–smart plug architecture should be understood as a layered framework rather than a fully validated tri-modal ADL recognition system. The empirical core evaluates the radar–thermal HAR branch, while smart plug telemetry is treated as a complementary branch for appliance-mediated IADL evidence. Full temporal integration of physical movement, posture context, and appliance use events remains for future work.

## 6. Conclusions and Future Work

This study evaluated low-cost ambient sensing for ADL monitoring in intelligent building environments. Under recording-grouped five-fold cross validation, the joint radar–thermal results show that the Hi-Link LD2450 sparse-track radar was competitive for binary motion recognition but was insufficient for more granular activity recognition. On the other hand, the thermal model using the Melexis MLX90640 32×24 array scored almost identically for binary motion recognition but outperformed radar in dynamic activity recognition. These findings suggest that the low-cost and sparse commercial radar is suitable for motion gating, and low-resolution thermal sensing enables posture and spatial information that is not present in radar tracking. The findings also argue that these characteristics are complementary; while multimodality provided a modest gain for binary motion, the results show that the values for multi-class activities were significantly higher than for both unimodal approaches. The separate smart plug branch of the proposed approach shows that XGBoost and random forest were the most promising machine learning models for appliance recognition. Analysis showed that XGBoost provided a stronger deployment tradeoff of file size and inference latency. In terms of features, ablation studies revealed that the proposed power factor features contribute useful information beyond the active power statistics that were most common in the related literature.

There were several scientific limitations during the experiments presented by this work. Firstly, the folds contained a single subject, and therefore future inter-subject validation is required towards generalisation of the proposed approach. Recording-grouped folds control data leakage, but leave-one-subject-out validation would provide a better overview of generalisation if data are collected from additional subjects. Second, all HAR recordings were collected in the same building with fixed sensor placements, and data from more dynamic environments could be collected in the future, such as with clutter, furniture, or sensor heights and angles as variables. Third, the reduced postural detection scores in the radar branch are specifically tied to the LD2450 track-level outputs and therefore not applicable to richer (and more costly) types of Doppler data from more advanced sensors. Finally, the appliance recognition dataset, despite being large in scale, is limited to the known appliance classes, and more data could be collected to extend the range of which appliances can be detected. Future work could therefore extend the evaluation with a larger participant panel (enabling LOSO validation) and further deployment across additional environments. The findings of this work show promise in the use of multimodal radar–thermal activity recognition and smart plug-based appliance recognition, and the next empirical step is to further fuse the three modalities towards a more integrated ambient monitoring system to support ambient assisted living in intelligent buildings.

## Figures and Tables

**Table 1:** Summary of the experimental room configurations.

Environment	Key Characteristic
E1	Empty room baseline
E2	Central inflatable bed
E3	Chair beneath sensors
E4	Bed with fall mat
E5	Dynamic chair placement
E6	Bed repositioned toward room edge

**Table 2:** Dataset composition, windowing parameters, and validation protocol for the radar–thermal HAR tasks.

Attribute	Binary Motion	Four-Class Posture and Motion
Subject	Single subject (S01)	
Environment	Six furniture configurations in one room	
Recording sessions	26	
Total duration	≈4.4 h (≈264 min)	
Window length	20 frames	
Window stride	5 frames	
Total windows	8114	7988
Classes	Moving; Stationary	Lying; Sitting; Standing; Walking
Windows per class	Moving: 2195; Stationary: 5919	Lying: 2282; Sitting: 1575; Standing: 2062; Walking: 2069
Minority class	Moving: 27.0%	Sitting: 19.7%
Validation protocol	Fivefold StratifiedGroupKFold grouped by recording identifier	

**Table 3:** Dataset composition, windowing parameters, and validation protocol for the smart plug appliance recognition task.

Attribute	Smart Plug Appliance Recognition
Total windows at W=60s	Laptop: 237,841; microwave: 4109; kettle: 7462; toaster: 723; idle: 2,802,934
Minority active class	Toaster, 723 windows at W=60s
Sensing device	MOKO MK117 smart plugs
Monitored channels	Active power, voltage, current, and scalar power factor recorded at 1 Hz
Task	Five-class appliance state recognition
Included classes	Laptop; Microwave; Kettle; Toaster; Idle/Off
Inclusion criterion	Appliance classes with at least three monitored instances are included. Single-instance appliances are excluded from the reported recognition task
Window lengths	W∈{10,60,300}s
Reported dataset count case	Counts are reported for the median window duration, W=60s
Input representation	16 statistical features extracted from active power (P) and power factor (PF)
Feature statistics	Mean, standard deviation, minimum, maximum, skewness, kurtosis, and range computed over each window
Preprocessing	Noise floor gating with $\tau_{P}=5\;W$ and consolidation of low-power states into a single idle/off class
Class distribution	Highly imbalanced, with Idle/Off dominating the natural distribution and Toaster forming the minority active class
Validation protocol	Chronological train, validation, and test partitions
Leakage control	A 300-s safety gap is inserted between adjacent partitions to reduce leakage from overlapping windows
Evaluation scope	Known-appliance temporal generalisation; not unseen-device or unseen-appliance class generalisation

**Table 4:** Winner summary for the unimodal pipelines. Accuracy, balanced accuracy, macro-AUC, and macro-$F_{1}$ are mean ± standard deviation over five recording-grouped folds.

Task	Modality	Winner	Accuracy	Balanced Acc.	Macro-AUC	Macro $F_{1}$
Binary motion	Radar	Transformer	0.910±0.017	0.880±0.043	0.951±0.022	0.882±0.034
Binary motion	Thermal	XGBoost	0.904±0.050	0.861±0.068	0.934±0.065	0.870±0.069
Four-class posture	Radar	Random forest	0.648±0.123	0.616±0.118	0.838±0.069	0.609±0.110
Four-class posture	Thermal	Random forest	0.794±0.047	0.778±0.053	0.936±0.027	0.775±0.053

**Table 5:** Radar machine learning baselines. Values are mean ± standard deviation over five recording-grouped folds.

Arm	Binary (Macro F1)	Four-Class (Macro F1)
LogisticRegression	0.834±0.079	0.574±0.083
RandomForest	0.853±0.082	0.609±0.110
MLP	0.848±0.083	0.572±0.096
XGBoost	0.851±0.079	0.600±0.098

**Table 6:** Radar deep learning baseline values are mean ± standard deviation over five recording-grouped folds.

Arm	Binary (Macro F1)	Four-Class (Macro F1)
MLP_on_features	0.851±0.073	0.567±0.077
CNN1D	0.873±0.048	0.539±0.090
LSTM	0.863±0.030	0.544±0.044
CNN_LSTM	0.851±0.057	0.533±0.039
Transformer	0.882±0.034	0.565±0.065

**Table 7:** Thermal machine learning baselines. Values are mean ± standard deviation over five recording-grouped folds.

Arm	Binary (Macro F1)	Four-Class (Macro F1)
LogisticRegression	0.849±0.057	0.750±0.054
RandomForest	0.860±0.071	0.775±0.053
MLP	0.860±0.067	0.773±0.054
XGBoost	0.870±0.069	0.724±0.087

**Table 8:** Thermal static-DL baselines. Values are mean ± standard deviation over five recording-grouped folds.

Arm	Binary (Macro F1)	Four-Class (Macro F1)
MLP_on_flat_frame	0.666±0.085	0.533±0.045
CNN2D_small	0.673±0.099	0.455±0.269
CNN2D_mid	0.761±0.070	0.608±0.120
CNN2D_deep	0.726±0.128	0.551±0.178

**Table 9:** Thermal temporal-DL baselines. Values are mean ± standard deviation over five recording-grouped folds.

Arm	Binary (Macro F1)	Four-Class (Macro F1)
Frozen2D_MeanPool	0.648±0.091	0.258±0.074
TemporalPool_CNN	0.734±0.142	0.557±0.203
Conv3D_small	0.689±0.100	0.492±0.229
ConvLSTM_small	0.839±0.061	0.732±0.079

**Table 10:** Modality comparison table.

Task	Radar Fixed Arm	Thermal Fixed Arm
Binary motion	Transformer 0.882±0.034	XGBoost 0.870±0.069
Four-class posture	RandomForest 0.609±0.110	RandomForest 0.775±0.053

**Table 11:** Fusion comparison.

Task	Candidate	Radar Arm	Thermal Arm	Macro $F_{1}$
Binary motion	Single radar	Transformer	—	0.882±0.034
Binary motion	Single thermal	—	XGBoost	0.870±0.069
Binary motion	Pair A	Random forest	XGBoost	0.873±0.067
Binary motion	Pair B	Transformer	XGBoost	0.895±0.039
Four-class posture	Single radar	Random forest	—	0.609±0.110
Four-class posture	Single thermal	—	Random forest	0.775±0.053
Four-class posture	Pair A/B	Random forest	Random forest	0.792±0.051

**Table 12:** Smart plug appliance recognition summary at the best 10-s window. Macro $F_{1}$ is reported as mean ± standard deviation over the repeated runs in the appliance benchmark.

Model	Window	Macro $F_{1}$	Footprint	Latency
Random forest	10 s	0.977±0.002	1.5 MB	38 ms
XGBoost	10 s	0.976±0.004	0.828 MB	0.4 ms
Two-layer MLP	10 s	0.743±0.009	51 kB	0.2 ms

## Data Availability

The smart plug appliance recognition dataset generated and analysed in this study is made publicly available to the research community at: https://www.kaggle.com/datasets/birdy654/ntu-ist-smart-plug-appliance-recognition-dataset (accessed on 3 July 2026).
